# A photovoltaic panel cleaning robot with a lightweight YOLO v8

**DOI:** 10.3389/frobt.2025.1606774

**Published:** 2025-10-31

**Authors:** Jidong Luo, Guoyi Wang, Yanjiao Lei, Dong Wang, Yayong Chen, Hongzhou Zhang

**Affiliations:** 1 College of Mechanical and Electrical Engineering, Tarim University, Alar, China; 2 Modern Agricultural Engineering Key Laboratory at Universities of Education Department of Xinjiang Uygur Autonomous Region, Alar, China; 3 Xinjiang Production and Construction Corps (XPCC) Key Laboratory of Utilization and Equipment of Special Agricultural and Forestry Products in Southern Xinjiang, Alar, China

**Keywords:** cleaning robot, photovoltaic station management, lightweight YOLO v8, deep learning enhancement, path planning

## Abstract

Cleaning PV (photovoltaic) panels is essential for a PV station, as dirt or dust reduces the effective irradiation of solar energy and weakens the efficiency of converting solar energy into free electrons. The inconsistent (cleaning efficacy) and unsafe (summarized voltage and current) manual method is a challenge for a PV station. Therefore, this paper develops a cleaning robot with PV detection, path planning, and action control. Firstly, a lightweight Mobile-VIT (Mobile Vision Transformer) model with a Self-Attention mechanism was used to improve YOLOv8 (You Only Look Once v8), resulting in an accuracy of 91.08% and a processing speed of 215 fps (frames per second). Secondly, an A* and a DWA (Dynamic Window Approach) path planning algorithm were improved. The simulation result shows that the time consumption decreased from 1.19 to 0.66 s and the Turn Number decreased from 23 to 10 p (places). Finally, the robot was evaluated and calibrated in both indoor and outdoor environments. The results showed that the algorithm can successfully clean PV arrays without manual control, with the rate increasing by 23% after its implementation. This study supports the maintenance of PV stations and serves as a reference for technical applications of deep learning, computer vision, and robot navigation.

## Highlights

1. YOLO v8 has been lightweighted with the Mobile-ViT for the cleaning robot.

2. The localizing and path planning algorithms were improved and validated in a computer environment.

3. Autonomous navigation and operation of robot prototypes in real-world environments.

## 1 Introduction

Cleaning photovoltaic (PV) panels for a PV station (solar power station) is a crucial prerequisite for achieving high efficiency and ensuring long-term, stable power generation (Azouzoute et al., 2021). Periodically cleaned at PV stations to prevent dust on the silicon crystal surface from blocking light or affecting the panel’s performance (Azouzoute et al., 2021). However, the traditional manual cleaning method is inefficient (time cost), variable (energy supply balance), and unsafe (voltage and current summation) for health (Mohd Nizam Ong et al., 2022). Automatic cleaning equipment and algorithms are likely to become a crucial means of replacing manual work and a key trend in future research (Şevik and Aktaş, 2022). In recent years, many PV cleaning studies have focused on the mechanisms of dust deposition (Patil et al., 2017; Hu et al., 2025), equipment development (Bergametti et al., 2014; Mariraj Mohan, 2016; Liu et al., 2021), and algorithm improvement (Sarode et al., 2023; Patil et al., 2017).

The dust deposition mechanism provides a comprehensive understanding of the movement, accumulation, and retention characteristics, which can provide a mathematical basis for effective cleaning. For example, Hu et al. (2025) used the correlation graph method to establish an adaptive relationship between the influencing factors and the deposition type for dust deposition on a PV panel. It developed an adaptive deposition model that accounts for capillary force, temperature, and humidity, resulting in a 39.5% reduction in error. Osmani et al. (2020) verified that the dust deposition mechanism is linked to the particle collision-adhesion mechanism and the environmental humidity through mechanical equilibrium and the adhesion law of energy. In a controllable particle size range and wind speed, properly increasing the inclination angle of photovoltaic panels can reduce dust accumulation. Mainstream studies on the dust particle deposition law by gravity (Bergametti et al., 2014; Mariraj Mohan, 2016), PV electrostatic adsorption of dust particles (Liu et al., 2021), the effect of airflow stress and flow velocity on dust deposition (Gao and Li, 2012; Qin et al., 2024), and dust accumulation by surface chemisorption (Hossain et al., 2022). For a power station, improvements in engineering techniques and systematic applications are also necessary.

A dust cleaning equipment for PV panels focuses on engineering effectiveness and technical applications (Osmani et al., 2020). For example, Habib et al. (2021) developed an auto-removing dust accumulation system on PV panels using an Arduino controller, a fan, and a soft cloth wiper, achieving 87% cleaning efficiency with no water usage. Chen Y. et al. (2023) developed an intelligent embedded hybrid system for cleaning PV panels using Linear Piezoelectric Actuators and Support Vector Machines, achieving 10%–30% recovery of dust and energy management parameters (error <5%). The hybrid system was evaluated in India’s tropical climate, achieving a steady 15%–28% increase in efficiency, along with a 40% reduction in operations and maintenance costs. Compared to stationary cleaning equipment, ground mobile robots (Antonelli et al., 2020; Li and Li, 2022) or aerial cleaning robots (Huang et al., 2025; Milidonis et al., 2023) offer higher flexibility, and a single machine can cover service work in multiple areas with better equipment and attachment economics. Aerial robots also need to consider the safety of low-altitude flight, the speed of single cleaning, and the necessary parameters, such as counterweight.

Whether a cleaning equipment is stationary or movable, on the ground or in the air, the application of intelligent algorithms has seen a trend and an integral part (Licardo et al., 2024). For example, Kshetrimayum et al. (2023) proposed a UAV (Unmanned Aerial Vehicle) for detecting, locating, and cleaning bird droppings in a PV station using an improved YOLO v7 (You Only Look Once), which calculates the dust distance and guides the cleaning equipment to clean efficiently. Chen B. R. et al. (2023) improved the fuzzy motion, rolling, and steering control for intelligent balance control and trajectory tracking, and found that the underdrive one-wheeled system can effectively pass various experimental scenarios, such as slope traversal and load disturbance. Yuan et al. (2024) noted that intelligent aerial robots have a broad range of applications in multiple fields, including both military and civilian contexts. The use of intelligent algorithms, such as deep learning, is of great benefit to the accuracy and work effectiveness of equipment robots.

Although many studies have discussed surface dust deposition patterns, the design and development of cleaning equipment, and detection methods for contaminated objects in PV station cleaning, there is a notable lack of reports on detecting PV panels through deep learning, planning paths through optimization algorithms, and building a robot to automate the cleaning of PV panels. This study enhanced the YOLO v8 network model to accurately detect PV panels, refined the DWA algorithm for path planning, and developed a robot system to implement the algorithm and perform PV station cleaning tasks.

## 2 Materials

### 2.1 Image dataset and data enhancement

**FIGURE 1:**
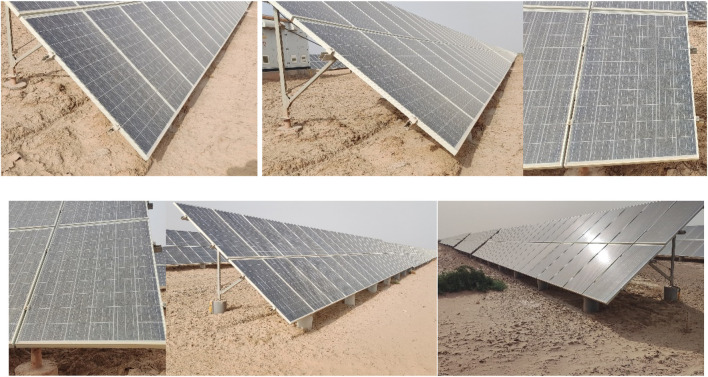
Partial image of the original dataset.

Processes such as training and testing of deep learning are performed on a ground-based image dataset (P-Pose, position and orientation). A typical PV station with multiple rows, pitted ground, and complex sunlight reflections was selected as the data acquisition site. During the data acquisition process, the robot was simulated with various shooting angles, oblique lateral, frontal views, high poses, backlight, and front light, and at different distances on 7 June 2023, as shown in Figure 1. The acquisition method is manual shooting acquisition, and the acquisition tool is a smartphone (Redmi K40S, Shenzhen, China, Xiaomi Technology Co., Ltd.), with a resolution of 4,000 × 1800. A total of 116 PV images were obtained as the original dataset, and the field dataset was constructed.

**FIGURE 2:**
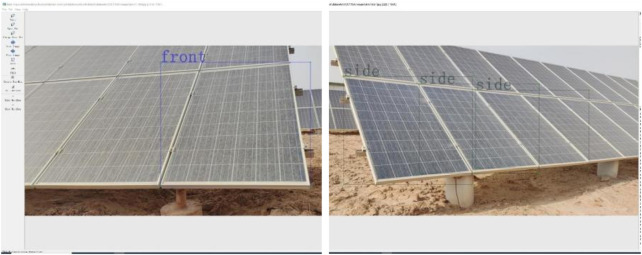
Data labeling results of two random images in the dataset.

The calibration process of the P-Pose dataset involves transforming the PV panel position and category information in the image into a computer-readable digital form. The complete dataset trained by the deep learning model for PV panel pose contains the PV pose category and the RoI (region of interest), which is used to obtain the coordinate position of the label to be recognized within the entire image. The labelled dataset in this folder is divided into one-to-one correspondences in the PV pose images. The dataset is labelled with the LabelMe app, which provides the “Create Rect-Box” rectangular shape. For a robot to start the cleaning, the labels are classified into “front” and “side”, as shown in Figure 2, simulating the front and oblique side positions from the corresponding machine viewpoints. Labels of the same type will be automatically assigned the same colour when the next labelling operation is performed. Due to the compact arrangement in the PV station and the characteristics of multiple PV panels visible from the same viewpoint, the same position is repeatedly labelled to enhance robustness. The parameters include ${class}_{id}$ , *x*, *y*, *w*, and *h*, where the ${class}_{id}$ denotes the class number of the target object. When ${class}_{id}=0$ , the labeled was denoted “side”, and ${class}_{id}=1$ denoted “front”. The *x*, and *y* are the horizontal and vertical offsets of the coordinates of the upper-left corner in the image. Then the *w* and *h* represent the width and height of the target label box. The four values are obtained after normalisation, i.e., the original data are mapped to the range of [0, 1] through a linear transformation for improved training and prediction.

**FIGURE 3:**
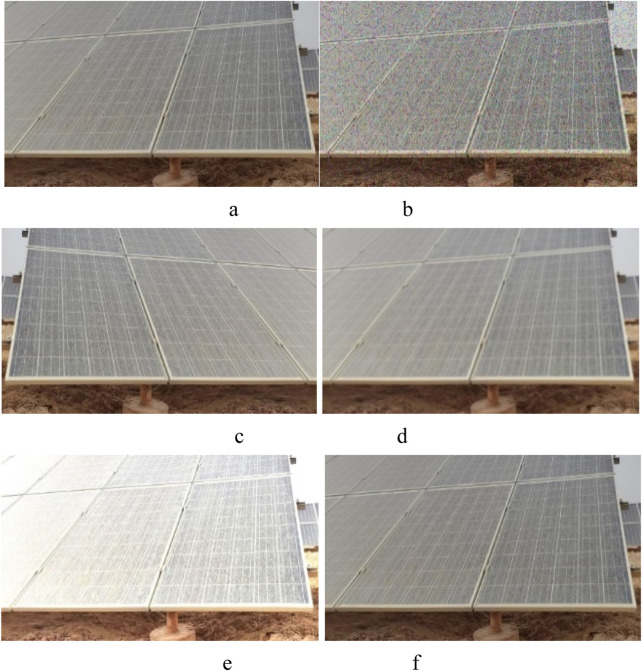
Examples of data augmentation. **(a)** A random RGB image data. **(b)** Pepper noise added to the a image. **(c)** Horizontal flip of the a image. **(d)** Gaussian noise added to the a image. **(e)** Brighten augmentation of the a image. **(F)** Dimed for the a image.

To improve its generalization ability and robustness, data augmentation techniques were employed to enhance the training data in this study. Firstly, the data is lossless compressed and normalized to the following format: resolution 950 × 428, RGB (Red/Green/Blue channels), and JPG (Joint Photographic Experts Group) file type. Then, the (0) no noise adding, (1) the Gaussian noise adding, (2) the Pepper noise adding, (3) the horizontal flipping, (4) the image blurring, (5) the luminance transformation, (6) the scaling, (7) the image translation, (8) and the rotation transformation were used to process all image in the dataset. All image examples were expanded to six times transformation (3–8) and three times noise levels (0, 1, 2) to obtain a 2,088-image dataset (6 × 3 × 116 = 2,088), as shown in Figure 3. To ensure the robustness and generalization ability of the model, all dataset samples are randomly divided into three sets, with a ratio of 8:1:1, resulting in 1,670 training set samples, 209 validation set samples, and 209 test set samples.

### 2.2 YOLO v8 structure and the performance evaluation parameters

**FIGURE 4:**
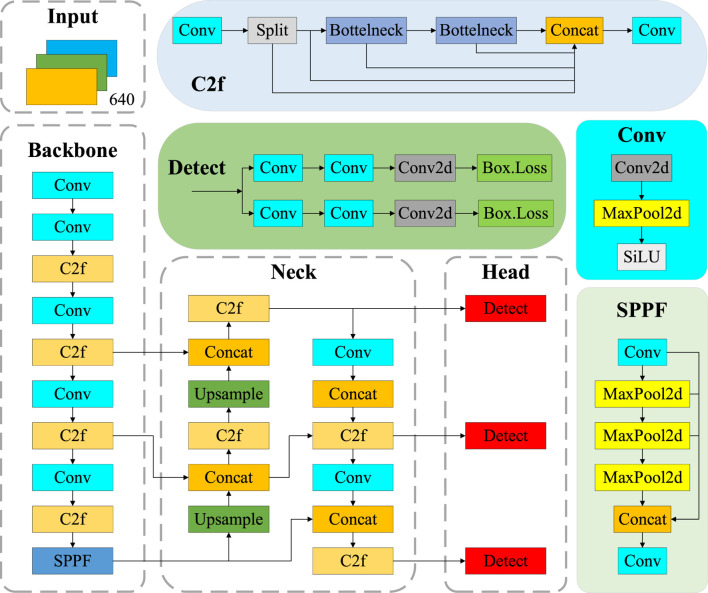
The YOLO v8 network model structure.

YOLO v8, developed by Ultralights, enhances efficiency and accuracy by building upon YOLO v6 and YOLO v7, which are primarily applied to tasks such as image classification, object detection, and instance segmentation. In this paper, we focus on the task of object detection. YOLO v8 consists of five models, including YOLO v8n, YOLO v8s, YOLO v8m, YOLO v8l, and YOLO v8x (Gamani et al., 2024). Considering the effect of model size, the YOLO v8n network model, which is compact yet highly accurate, is chosen in this paper. As shown in Figure 4, the YOLO v8n model’s detection network is primarily composed of four key components: Input, Backbone, Neck, and Head.

The Input part is the data preprocessing component of the model, which includes multi-scale detection and normalization of pixel values to the range of [0, 1]. It helps improve the model’s ability to manage different image brightnesses and contrasts better. Batch processing enables the Backbone model to process multiple images simultaneously. The Backbone contains the Conv, C2f (Cross Stage Partial Network 2 with Focus), and SPPF (Spatial Pyramid Pooling-Fast) modules (Liu and Zhou, 2024). The Conv module is primarily used for convolution and combines Batch Normalization (BN) and Sigmoid Linear Unit (SiLU) activation functions to process the input image. In addition, the SPPF structure converts an arbitrarily sized feature mapping into a fixed-size feature vector. The neck structure combines different layers of feature mapping to enhance detection performance, thereby constructing a feature pyramid. It helps the model to process multi-scale information more efficiently and perform learning and inference (Liu et al., 2018). The Head is the detection part of the target detection model, which employs strategies such as multi-stage prediction and cross-feature-graph linking to fuse multiple feature mapping outputs from the neck according to specific rules, thus obtaining a global feature vector box. Loss is a technique used to compute the position of the BBR in target detection and the size regression loss, which consists of complete IoU (Intersection over Union) Loss and Distribution Focal Loss. Complete IoU Loss, as the BBR loss function, considers the position, size, aspect ratio, and angle information of the box to measure the similarity between the predicted box and the real box more comprehensively. On the other hand, Distribution Focal Loss is used to suppress positional regression inaccuracies, which causes the model to pay more attention to samples that are difficult to regress, thereby effectively improving the positional BBR. The output Complete IoU is obtained by weighing the location and size difference factors, which in turn calculate the regression loss by measuring the difference in Complete IoU values between the predicted and real boxes.

The model was constructed on computer workstations in the on-campus laboratory, the experimental environment, with 64-bit Windows 10 operating system for training and validation, the computer hardware system configuration includes: the central processor is an Intel of Xeon ® Silver 4210R CPU @ 2.40 GHz; the graphics card is an NVIDIA GeForce RTX 3060Ti; and 64 GB of operating memory. The programming and deployment of the YOLO v8 algorithm were conducted on the PyCharm 2023 platform using Python 3.8.17 as the programming language. The training was accelerated by using CUDA 11.8, and the network framework training was also conducted based on the deep learning framework PyTorch 2.0.0.

**FIGURE 5:**
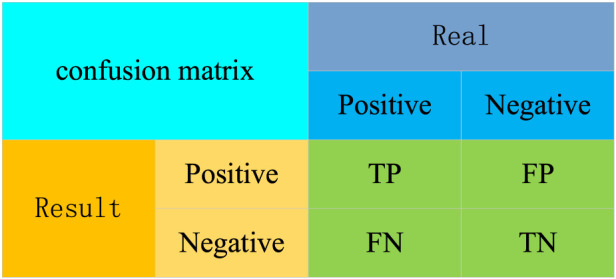
The confusion matrix.

For the evaluation of the results models, the confusion matrix, precision (*P*), recall (*R*), mean average precision (*mAP*), F1 score (*F1*), number of floating-point operations (*GFLOPs*), and Frame Per Second, (*FPS*, unit fps) were used as evaluation metrics for the PV panel position detection model. In this case, the confusion matrix is used to evaluate the performance of the binary classification model, comparing the model’s prediction results with the actual results to produce quantitative statistics for the four cases. The predicted and the actual results are categorized as positive and negative cases. These predictions can be represented in a confusion matrix (Figure 5).

The confusion matrix in (1) True Positives (*TP*): the samples number in which the model correctly predicts positive cases as positive; (2) True Negatives (*TN*): the samples number in which the model correctly predicts negative cases as negative; (3) False Positives (*FP*): the samples number in which the model incorrectly predicts negative cases as positive cases; (4) False Negatives (*FN*): the samples number in which the model incorrectly predicts positive cases as negative cases. Additionally, the *P* refers to the proportion of positive samples that are predicted correctly, as shown in Equation 1.

$$Precision=\frac{\text{TP}}{\text{TP}+\text{FP}}$$ (1)

The *R* can be calculated as the proportion of all targets correctly predicted, as in Equation 2.

$$Recall=\frac{\text{FP}}{\text{TP}+\text{FN}}$$ (2)

The *mAP* evaluates the performance in the target detection task by drawing the *P-R* curve and calculating the area under the curve. This study also used the *mAP* to assess the result, which is a balance between *P* and *R*, as the evaluation index for the detection model. The *mAP* can more accurately assess the performance of the model by considering the sorting effect of the model on distinct categories in the target detection. *mAP* is directly proportional to the performance of the algorithm model, and it can reflect the model’s performance more comprehensively and objectively. The *m* denotes the average, and the number after denotes the threshold for determining whether a sample is positive or negative in terms of *IoU*. The *mAP* is the average accuracy of the *n* categories, calculated as shown in Equation 3.

$$mAP=\frac{1}{n}\sum_{i=1}^{1}\int_{0}^{1}Precision\left(Recall\right)d\left(Recall\right)$$ (3)

The *F1* is the reconciled average of the detection rate and *R*, so the *F1* curve is usually used to compare the performance of different models. The *F1* can range from [0,1], and the larger, the better performance of the model, as shown in Equation 4.

$$F1=2\times \frac{Precision\times Recall}{Precision+Recall}$$ (4)

The *GFLOPs* measures the complexity of the model; higher values typically indicate that the model requires more computational resources for inference, resulting in longer inference times. On the contrary, a lower value indicates that the model is less computationally intensive and can complete the reasoning process faster. The detection frame rate refers to the *FPS* at which the processed image is displayed. A higher *FPS* means that the system can process images or videos faster, resulting in a smoother display or faster detection. The *FPS* is affected by both the algorithm weights and the hardware configuration of the experimental equipment.

### 2.3 The design of the robot

**FIGURE 6:**
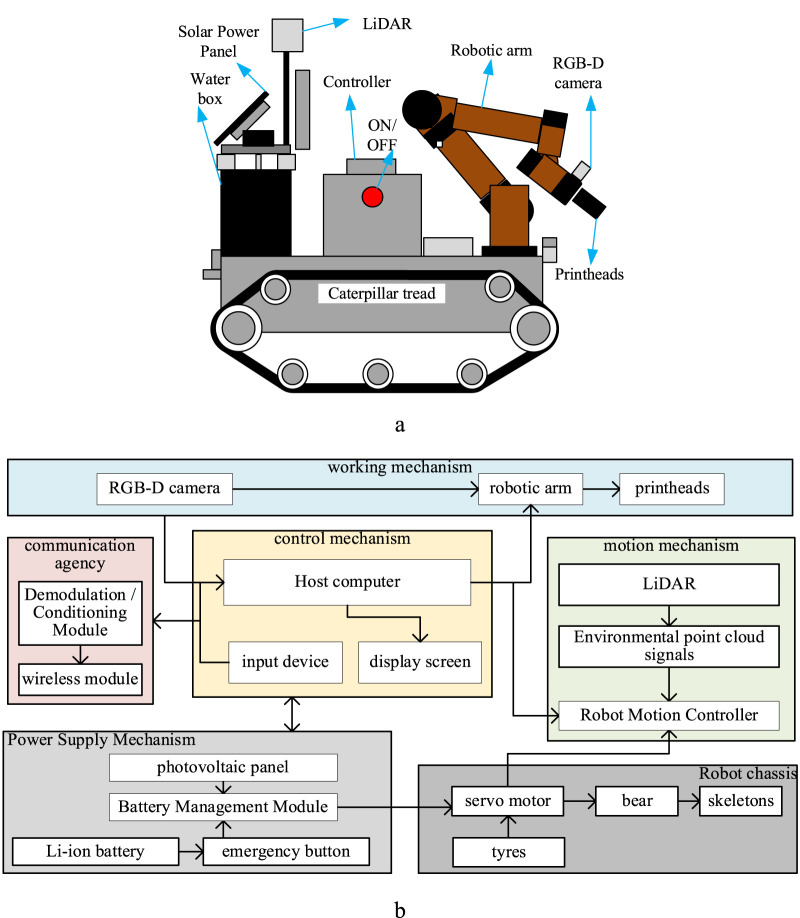
Primary structure of the robot. **(a)** The design of the robot. **(b)** The structural diagram of the robot.

The overall structure of the robot was designed independently, as shown in Figure 6, including the power supply structure, robot chassis (Bunker mini), mechanical arm (JAKA C4), control mechanism, motion mechanism, communication mechanism, camera, and working mechanism (water box, pumps and nozzles). Among them, the Li battery (24 V/10,000 mA h) and power management module of the power supply mechanism provide voltage to the entire robot. The emergency button is directly connected to the battery, and the PV panel serves as an additional power source for charging the battery. The host computer and input devices, such as display outputs and keyboards, form an independent control mechanism that is connected to other mechanisms to send control signals and execute task control processes. The robot motion mechanism serves as an independent motion control centre with the Robot Operating System (ROS 2 Foxy Fitzroy) and is connected to the host computer via communication. This independent control centre receives point cloud signals from the LiDAR (Light Detection and Ranging) and outputs control signals for the motion to the chassis mechanism, enabling the entire robot to move. The chassis is the skeleton, bearing, motor, and tire parts that provide the structural force to achieve the movement of the whole robot. The working mechanism comprises a robotic arm, an RGB-D (RGB-Depth) camera (Astra+ with Orbbec SDK), and a nozzle part. The camera acquires image information in real-time, and the nozzle outputs high water pressure (90 ± 10 kPa), allowing the robotic arm to adjust the position and angle of the output. The communication mechanism prepares the entire robot for networking with other robots and the whole cleaning task.

## 3 Methods

### 3.1 YOLO v8 structure and cost function improvement approach

To further improve the accuracy and efficiency of YOLO v8, the box cost function has been optimized to enhance the training and testing of the network, enabling the PV panels cleaning robot to identify variable PVs in the mobile view. Among them, Mobile-ViT is further optimized based on the efficient and lightweight visual ViT model and Transformer model (Mehta et al., 2021), which is commonly used in computing resource and storage space-constrained recognition and detection environments for outdoor deployments of movable equipment.

**FIGURE 7:**
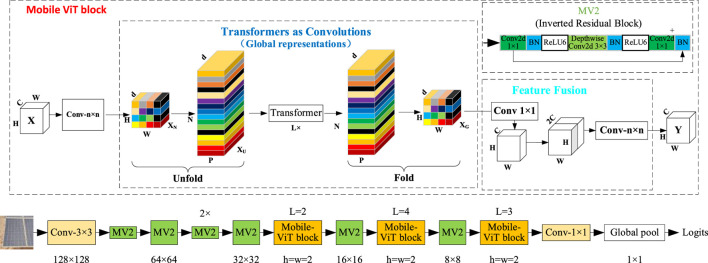
Mobile-ViT network structure.

Figure 7 provides a schematic of the improved Mobile-ViT model, including its detailed components: Mobile-ViT block, Transformer, MV2, and feature fusion. Each Block in the Mobile-ViT module consists of MHSA (Multi-Head Self-Attention) layers and FFNN (Feed-Forward Neural Networks) layers stacked alternately. The feature mapping is generated by a convolutional module of size n × n for local feature modeling, followed by a convolutional layer with a 1 × 1 convolutional kernel to adjust the channel number. This is then followed by global feature modelling through the Unfold, Transformer, and Fold structures, in that order. Then, a convolutional layer with a 1 × 1 convolutional kernel size is used to adjust the channel size to the original size, followed by shortcut branching with the original input feature mappings spliced by the channel concept. Finally, a convolutional layer with a convolutional kernel size of n × n is used for feature fusion to obtain the final output, as shown in Equation 5.

$$X_{T}\left(p\right)=\text{Transforme}\left(X_{U}\left(p\right)\right),p\in \left[1,p\right]$$ (5)

Where the *N* is the number of spreading picture blocks, the *p* is the size of the picture blocks; the *W* and *H* are the width and height of the spreading picture blocks. In the Transformer module, Self-Attention is used to learn the correlation between various locations in the image, thereby fully understanding the inner structure of the image features. Self-Attention calculates each token by considering the correlation with all other tokens using the formula, as shown in Equation 6.

$$Attention\left(Q,K,V\right)=softmax\left(\frac{QK^{T}}{\sqrt{d_{k}}}\right)V$$ (6)

Where *T* denotes the length of the sequence, *dk* denotes the dimension of *K* (*dq*, and *dv* are for *Q*, and *V*). Before calculating SoftMax, the weight matrix is multiplied by the *d*_*k*_ *= -0.5*. After completing the calculation of *XX* ^*T*^, the variance of the elements in the matrix becomes large, causing the SoftMax distribution to become extremely steep, which affects the stable calculation of the gradient, as Equation 7. The previously computed similarity can be normalized, and the variance can be adjusted to *1/N*, which decouples the steepness of the SoftMax distribution from *d*. Before training and testing, the class weight was set as an inverse proportion of the example to the classes balance, as Chen Y. et al. (2023) reported. Therefore, the stability of the gradient can be maintained during the training process, allowing the Self-Attention mechanism to be implemented and the Self-Attention feature extraction to be completed.

$$Y=softmax\left(XX^{T}\right)X$$ (7)

Finally, the contextual dependencies between local and global blocks are established through the channel fusion and recovery operations, as shown in Equation 8.

$$X_{G}=\xi \left(X_{N},X_{U}\right)$$ (8)

Where the *X*_*N*_ denotes the input local feature block, *X*_*U*_ denotes the expanded local feature block, *X*_*G*_ denotes the fused feature block, and *ξ* denotes the feature-weighted fusion operation. The feature reduction of the channel is performed using multi-scale convolution, and the extraction of positional features for PV panels is achieved through a feature fusion structure.

In addition, considering that YOLO relies on the BBR (Bounding Box Regression) module to determine the location of objects (Girshick et al., 2015), during the training process. The BBR loss function is design as MPDIoU. The core design idea of the MPDIoU is to define two metrics, the centroid distance discrepancy metric (MD) and the target box width-height discrepancy metric (PD). In this case, the MD is calculated by determining the Euclidean distance (ED) between the centroids of the two target boxes and then normalising it to the range of [0, 1], as shown in Equation 9.

$$MD=\frac{{\left|\left|c_{1}-c_{2}\right|\right|}_{2}}{\sqrt{w_{1}^{2}+h_{1}^{2}+w_{2}^{2}+h_{2}^{2}}}$$ (9)

Where the *c*_*1*_ and *c*_*2*_ denote the coordinates of the centre points of the two target boxes. The *w*_*1*_ and *h*_*1*_ denote the width and height of the first target box. And the *w*_*2*_ and *h*_*2*_ denote the width and height of the second target box, by the sum of the ED between the coordinates of the upper left and lower right corners of the two target frames and normalizing this distance to [0,1], as Equation 10.

$$PD=\frac{{\left|\left|p_{tl}^{1}-p_{tl}^{2}\right|\right|}_{2}+{\left|\left|p_{br}^{1}-p_{br}^{2}\right|\right|}_{2}}{\sqrt{w_{1}^{2}+h_{1}^{2}+w_{2}^{2}+h_{2}^{2}}}$$ (10)

Where the $p_{tl}^{1}$ and $p_{tl}^{2}$ denote the coordinates of the upper left corner of the two target frames. The $p_{br}^{1}$ and $p_{br}^{2}$ denote the coordinates of the lower right corner of the two target frames, as shown in Equation 11.

$$MPD=\max\left(MD,PD\right)$$ (11)

The overlap metric MPD and traditional IoU are weighted and fused to obtain the MPDIoU , as shown in Equation 12.

MPDIoU=IoU+α·MPD (12)

where α is a hyperparameter used to control the weighting between the two metrics. Define the BBR loss function LMPDIoU based on MPDIoU according to the definition of MPDIoU, as Equation 13.

$$L_{MPDIoU}=1-MPDIoU$$ (13)

Replacing class Bbox Loss and class YOLO v8 Detection Loss in the detection HEAD module with class $L_{MPDIoU}$ similarity comparisons between the target boxes make it more applicable to overlapping BBR, improving the speed of convergence and the accuracy of the regression results. The method comprehensively considers the centroid distance difference and width-height difference between PV positional, thus providing a more comprehensive and accurate result.

### 3.2 Algorithm improvement and simulation for robot path planning

The previous deep learning target recognition results also need to be integrated with the path planning algorithm to determine the working path, enabling the robot to achieve continuous automatic operation. The iterative process of the A* algorithm is enhanced by self-regulated search and path smoothing rules, and local optimization is applied to optimize the real-time performance of the A* algorithm (Li and Zhang, 2023). Its core principle is to find the optimal heuristic choice by comparing the relationship between the heuristic cost and the global cost, as expressed in Equation 14.

$$f\left(n\right)=g\left(n\right)+h\left(n\right)$$ (14)

Where the n denotes the identification of the current scene-searching locus. The $f\left(n\right)$ denotes the minimum surrogate value from the robot’s initial locus to the target locus. The $g\left(n\right)$ denotes the actual minimum cost for the robot to travel from the initial locus to the current locus n . And the $h\left(n\right)$ is a heuristic function representing the expected cost for the robot to travel from the current locus n to the target locus. The unobstructed travelling path of two points at the current and target loci in the Cartesian coordinate system.

$$h\left(n\right)=\sqrt{{\left(x_{1}-x_{2}\right)}^{2}+{\left(y_{1}-y_{2}\right)}^{2}}$$ (15)

The improved A* algorithm used in this paper is capable of automatically adjusting the weighting coefficients of the heuristic function $h\left(n\right)$ , during the path planning process. Equation 15 is based on the addition of the self-adjusting weighting coefficient formula, denoted as Equation 16.

$$f\left(n\right)=g\left(n\right)+w\left(n\right)\cdot h\left(n\right)$$ (16)

where $w\left(n\right)$ denotes the self-adjustment weight coefficient and $w\left(n\right)\geq 1$ .

Considering that the PV panel cleaning robot utilises a tracked mobile chassis, the steering process requires multiple speed adjustments, which increases the difficulty of controlling the chassis movement and reduces the robot’s efficiency. The improved A* algorithm in this paper also requires further smoothing and optimization using Bessel curves, as shown in Equation 17.

$$B\left(t\right)=\sum_{i=0}^{n}\left(\frac{n}{i}\right){\left(1-t\right)}^{n-1}t^{i}P_{i}$$ (17)

Where the $\frac{n}{i}$ denotes the binomial coefficients. The *t* denotes a parameter and satisfies, where t≥0 . The $P_{i}$ denotes a control locus. The *n* is proportional to the degree of smoothing of the path. And the $P_{0},P_{1},\ldots ,P_{n}$ denote the control points, and the planning result of the path that passes through the locus $P_{0}$ and to $P_{n}$ .

Furthermore, considering that the robot’s operating environment during motion is dynamic, it is necessary to investigate the need for localized consideration of real-time obstacle avoidance in the capability process. Therefore, the dynamic window of the robot was enhanced by its dynamics and inertia, and feasible arc trajectories were generated by selecting appropriate velocities. Various constraints can be added as needed. Assuming that the robot’s traveling trajectory can be considered a straight line during a very short sampling interval, the increment of the robot’s displacement in the global coordinate system is now *∆t*, as shown in Equation 18.

$$\left\{\begin{array}{l} x\left(t\right)=x\left(t\right)\cdot v\left(t\right)\cdot ∆t\cdot \cos\theta \\ y\left(t\right)=y\left(t\right)\cdot v\left(t\right)\cdot ∆t\cdot \sin\theta \\ \theta \left(t\right)=\theta \left(t\right)+\omega \left(t\right)\cdot ∆t \end{array}\right.$$ (18)

Where the *x(t)* is the position component in the X direction of the world XOY axis, and the *y(t)* is the position component in the Y direction. The *θ(t)* is the direction angle. The *v(t)* is the line velocity. And the ω *(t)* is the angular velocity. Therefore, based on the limitation of the robot acceleration, the sampling space of the velocity at this time is given by Equation 19.

$$\begin{array}{ll} V_{a}= & \left(v,\omega \right),v\in \left[v_{c}-a_{vmax}∆t,v_{c}+a_{vmin}∆t\right],\\ & \;\omega \in \left[\omega_{c}-a_{\omega \max}∆t,\omega_{c}+a_{\omega \min}∆t\right] \end{array}$$ (19)

Where the $v_{c}$ and $\omega_{c}$ denote the linear and angular velocities of the robot at the current moment, as shown in Equation 20.

$$V_{z}=\left\{\left(v,\omega \right)|v\leq \sqrt{2d\left(v,\omega \right)\cdot a_{\omega \max}},\omega \leq \sqrt{2d\left(v,\omega \right)\cdot a_{\omega \max}}\right\}$$ (20)

Where the $a_{\omega \max}$ and $a_{\omega \min}$ denote the maximum and minimum values of the linear acceleration of the robot. The $a_{\omega \max}$ and $a_{\omega \min}$ denote the maximum and minimum values of the angular acceleration. The $d\left(v,\omega \right)$ denotes the distance between the robot and the nearest obstacle in the map, and only when the condition is satisfied will the robot not collide with the obstacle. The sampling speed of the robot must collectively satisfy the above constraints, i.e., the velocities of the robot, shown as Equation 21.

$$V=V_{l}\cap V_{a}\cap V_{z}$$ (21)

After calculating the robot’s traveling paths at different operational velocities, it is necessary to select the optimal one from these paths. The trajectory evaluation function evaluates all the predicted paths under the velocity sampling space. Then it chooses the velocity corresponding to the scored optimal travel path as the next velocity state. The mathematical formulation of the trajectory evaluation function is as Equation 22.

$$F\left(v,w\right)=\sigma \left(\alpha \cdot heading\left(v,\omega \right)+\beta \cdot dist\left(v,\omega \right)+\gamma \cdot velocity\left(v,\omega \right)\right)$$ (22)

Where α , β , γ denote the weight coefficients of the corresponding functions, respectively. The $heading\left(v,\omega \right)$ denotes that the robot reaches the end of the travelling path with the currently set sampling speed $\left(v,\omega \right)$ , and the angle Δθ between the robot’s facing and the target at this moment, and the degree of this angle is inversely proportional to the scoring. The $dist\left(v,\omega \right)$ denotes that the robot’s facing is the same as the nearest robot on the current traveling path. The $dist\left(v,\omega \right)$ represents the distance Δl between the robot and the nearest obstacle in the current travelling path, which is proportional to the score, and is set to a constant value assuming that there is no obstacle in this travelling path. The $velocity\left(v,\omega \right)$ represents the running linear velocity $v_{c}$ of the robot, and this value is proportional to the score.

### 3.3 Mapping and localization of robots in outdoor environments

Although the robot’s work path functions were discussed in the previous section under simulated conditions, actual robot work requires a detailed consideration of the entire process, including localization, spatial transformations, and testing of the actual work effect. Therefore, the Gmapping algorithm (Cuenca et al., 2023; Grisetti et al., 2005) is used, which has been improved for map construction, and the improved AMCL (Adaptive Monte Carlo Localization) algorithm, which primarily involves initialization, particle weight sampling, weight computation, resampling, and map updating. Among them, initializing particles means setting the weight of each particle to the average weight (w), as shown in Equation 23, so that *N* particles are initialized.

$$w_{0}^{\left(i\right)}=1/N$$ (23)

Particle weight sampling refers to relying on the data model acquired by the sensors to approximate the actual state of the mobile robot. Weight computation refers to the calculation of weights using Bayesian probability formulas in the traditional application of the RBPF algorithm. In this paper, the sensor observation data $Z_{s}$ is integrated into the proposed distribution, which focuses the sampling process on the region with the highest likelihood of observation, thereby improving sampling efficiency and accuracy, as shown in Equation 24.

$$w_{s}^{\left(i\right)}\approx w_{s-1}^{\left(i\right)}\cdot \sum_{j=1}^{k}P\left(Z_{s}|m_{s-1}^{\left(i\right)},X_{j}\right)P\left(X_{j}|X_{s-1}^{\left(i\right)},U_{s-1}\right)=w_{s-1}^{\left(i\right)}\cdot \eta^{\left(i\right)}$$ (24)

Resampling: scanning the surroundings through the sensors, scenarios with sparse localization features and high similarity may be encountered, which can skew the particle weights towards homogenization, resulting in most particles deviating from the actual state. In the actual calculation process, the particle weight dispersion metric $N_{\text{eff}}$ can be Equation 25.

$$N_{eff}=\frac{1}{\sum_{i=1}^{N}{\left(w_{s}^{\left(i\right)}\right)}^{2}}$$ (25)

Where the *N* denotes the number, and the $\sum_{i=1}^{N}{\left(w_{s}^{\left(i\right)}\right)}^{2}$ denotes the particle weight gap. At this point, resampling is required to replace the previously sampled particles. This strategy enhances the positioning accuracy of the particles, resulting in more accurately generated maps. Map update: The optimal particles are selected based on the particle weight sizes in the particle set $X_{s}^{\left(I\right)}$ , which determines the best trajectory for the mobile robot. Then, the map is updated in real-time based on this trajectory using sensor observations from LiDAR, and the constructed map is stored, thus completing the entire SLAM map-building process as described in Equation 26.

$$P\left(X_{1∶s},m|Z_{1∶s},U_{1∶s-1}\right)=P\left(m|X_{1∶s},Z_{1∶s}\right)\times P\left(X_{1∶s}|Z_{1∶s},U_{1∶s-1}\right)$$ (26)

Where $P\left(X_{1∶s}|Z_{1∶s},U_{1∶s-1}\right)$ denotes the joint estimation of the robot’s motion position, i.e., the robot’s localization, using the odometer control information and the sensor observation information, and $P\left(m|X_{1∶s},Z_{1∶s}\right)$ denotes the computation of the robot’s motion position in combination with the observation information for the computation and updating of the environment feature map.

Since then, the localization technique used has been AMCL, based on the PFA (Particle Filter Algorithm) [63]. In the process of calculating the particle weights, two additional data points need to be tracked, i.e., the mean value of the long-term change of the particle weights $w_{slow}$ and the mean value of the short-term change of the particle weights $w_{fast}$ , as shown in Equation 27.

$$\left\{\begin{array}{l} w_{fast}=w_{fast}+w_{fast}\left(w_{avg}-w_{fast}\right)\\ w_{slow}=w_{slow}+w_{slow}\left(w_{avg}-w_{slow}\right) \end{array}\right.$$ (27)

In the particle resampling phase, the algorithm performs random sampling of arbitrary particles to increase the number of random particles. The cleaning robot can accurately localize when the short-term variation mean $w_{fast}$ is equal to or greater than the long-term variation means $w_{slow}$ , when the particle population tends to converge. However, suppose the average measurement probability of each particle in the particle set decreases. In that case, the robot may encounter localisation problems, at which point random particles need to be added to resolve the issue, as shown in Equation 28.

$$\max\left\{0,1-w_{fast}/w_{slow}\right\}$$ (28)

To optimize the computational speed of the algorithm, AMCL employs KLD (Kullback-Leibler Divergence) sampling. This sampling method determines the size of the particle ensemble by calculating the distance between the approximation of the particle filter and the actual probability distribution, as in Equation 29.

$$M_{T}=\left(k-\frac{1}{2\alpha }\right){\left(1-\frac{1}{9\left(k-1\right)}+\sqrt{\frac{2}{9\left(k-1\right)\beta }}\right)}^{3}$$ (29)

Where the $M_{T}$ is the particle set size; *k* is the extent to which the particle subset covers the global map; *α* is the error threshold between the approximate probability distribution and the proper distribution; and *β* is the points on the normal distribution.

## 4 Result

### 4.1 YOLO v8 improvement results for PV panel detection

Table 1 presents the results of training and comparing the improved YOLO v8n-PP with 5 other similar deep learning models, including YOLO v5s, YOLO v7, YOLO v8s, and YOLO v8n, under the same datasets and training conditions. Under the condition of keeping the PV panels positional dataset, model hyperparameters, and training parameters consistent, it can be seen from the table that the YOLO v8n-PP model performs best in all performance metrics, and its seven algorithmic model evaluation metrics are significantly better than those of the other algorithms. On the contrary, the YOLO v5s model performs worse in all performance metrics, due to its use of a shallower feature extraction network. As a result, most performance evaluation metrics show lower levels. The results of this experiment demonstrate the superiority of the YOLO v8n-PP model in detecting the positional attitude of the PV panels.

**TABLE 1:** Comparative experiments of different algorithmic models.

Model	*P* (%)	*R* (%)	*mAP*@0.5 (%)	*F1* (%)	*GFLOPs* (t)	*mAP*@0.5:0.95 (%)	*FPS* (fps)
YOLO v5s	90.31	81.64	88.07	88	15.8	80.87	76
YOLO v7	89.13	82.24	85.93	87	11.6	82.65	87
YOLO v8s	90.37	82.31	88.22	86	28.3	87.63	157
YOLO v8n	91.43	83.33	88.95	87	8.2	86.63	126
YOLO v8n-PP	**94.88**	**89.11**	**93.36**	**93**	**6.1**	**91.08**	**215**

Note: Bolded data are optimal values.

**FIGURE 8:**
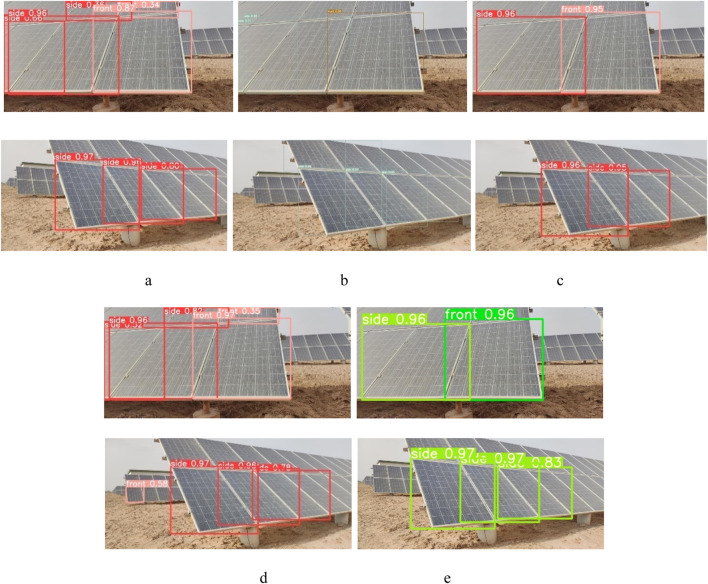
Comparison results of different algorithms. **(a)** Output of YOLO v5s. **(b)** Output of YOLO v7. **(c)** Output of YOLO v8s. **(d)** Output of YOLO v8n. **(e)** Output of YOLO v8n-PP (this paper).

To compare the recognition effect of different algorithm models on PV panel orientation more graphically, the optimal training weight file for each algorithm model is used to visualise and analyse the test set. The visualization results are shown in Figure 8. In this experiment, pictures with overlapping PV backgrounds and containing two types of orientations are selected as the objects of comparison. The comparative analysis reveals that the recognition accuracy of YOLO v8n surpasses that of the previous three algorithms. However, when dealing with PV panels positioned in overlapping backgrounds, the algorithm may appear to recognise PV targets that are heavily occluded, resulting in cluttered recognition target frames. It may also appear to recognize overlapping back-row PV panels. This phenomenon may cause serious interference with the robot, potentially preventing it from conducting the cleaning operation correctly. The YOLO v8n-PP further enhances the comprehensive performance of target detection and simultaneously enables accurate detection of the current PV panels to be cleaned under an overlapping background. This allows the cleaning robot to accurately adjust the path command during the autonomous navigation stage, ensuring smooth operation.

**FIGURE 9:**
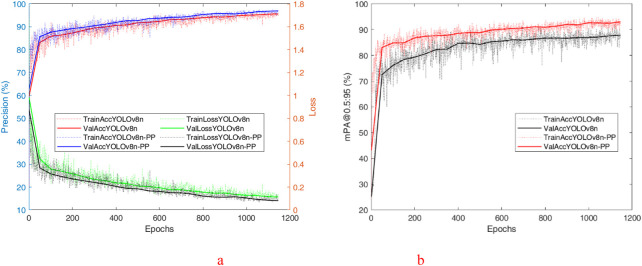
The training result of the YOLO v8n and YOLO v8n-PP. **(a)** The training *P*, Loss of the YOLO v8n and YOLO v8n-PP. **(b)** The training *mPA@50:90* of the YOLO v8n and YOLO v8n-PP.

To evaluate the performance of the improved algorithm more intuitively, the *P*, *R*, and *mAP@0.5* of the YOLO v8n and YOLO v8n-PP models are shown in Figure 9 during the training process for comparison. Figure 9 shows the visual comparison of *P* between YOLO v8n and YOLO v8n-PP models during the training process. According to the analysis of the experimental data, the YOLO v8n algorithm has a low initial *P* in the first 100 rounds of deep learning. It fluctuates with the increase in the number of rounds in deep learning. It is only after the 100th round that the *P* stabilizes and remains above 90%. However, until the end of training, the curve still exhibits significant fluctuations, indicating that the *P* is not stable enough. In contrast, the YOLO v8n-PP algorithm has an initial *P* about 10% higher relative to YOLO v8n in the first 100 rounds of deep learning, and the *P* grows more smoothly as the number of deep learning rounds increases. Before the 100th round, the *P* has reached a steady state, which persists until the end of training, and the *P* curve remains stable, consistently higher than that of YOLO v8n.

Demonstrates the YOLO v8n and YOLO v8n-PP models in the training process of *mAP@0.5* visual comparison. According to the analysis of the experimental data, YOLO v8n′s *mAP@0.5* remains volatile and has not yet reached a stable state at the end of training. Still, YOLO v8n-PP of *mAP@0.5* is larger than YOLO v8n and shows a smoother curve, which flattens out at the late stage of training. From the visualization results, the superiority of the YOLO v8n-PP model extends beyond numerical level advantages; the stability of its recognition ability is also crucial. The stability of its recognition ability is excellent in both the training and testing phases, consistently providing high-quality recognition results. This stability enables the model to remain efficient and accurate when managing complex tasks. Due to this, the YOLO v8n-PP model demonstrates excellent applicability when performing PV position recognition tasks on physical devices. Its stable recognition capability and excellent numerical performance enable the model to effectively cope with a variety of complex environments and conditions, thus providing great convenience in practical operations.

To verify the effectiveness of the improvement algorithm, three improvement YOLO v8n models were built for ablation-style experiments, including YOLO v8n-M, YOLO v8n-MPD, and YOLO v8n-PP. Among them, YOLO v8n-M is a lightweight model suitable for mobile devices, replacing Mobile-ViT as the Backbone. YOLO v8n-MPD is to replace class Bbox-Loss and class v8 detection loss in the detection HEAD module with the BBR loss function class LMPD-IoU-Loss. finally, YOLO v8n-PP is to replace class LMPD-IoU-Loss with class LMPD-IoU-Loss (this study model). Position recognition is a crucial visual task, and the original YOLO v8n has demonstrated strong reliability in this field. By analyzing the results of the ablation-style experiments in Table 2, the original YOLO v8n shows strong reliability in PV panel bit-position recognition. The initial deep learning using the unimproved YOLO v8n achieved 91.43%, 83.33%, 88.95%, 87%, and 126 fps fo*r P, R, mAP@0.5, F1*, and *FPS*, respectively. The excellent performance of these evaluated metrics highlights the potential of the YOLO v8n algorithm for PV bit-posture recognition. However, the algorithm still suffers from problems in the evaluation of detection metrics, such as low *F1*, *mAP@0.5:0.95*, and *GFLOPs*. Despite the model’s overall superior performance, there is still room for improvement in the algorithm’s metric performance in the field of target detection. Therefore, there is a need to continue exploring and optimizing YOLO v8n to enhance its performance in tasks such as PV position recognition.

**TABLE 2:** Results of YOLO v8 ablation-style experiment.

Model	Mobile-ViT	*MPD* *IoU*	*P* (%)	*R* (%)	*mAP*@0.5 (%)	F1 (%)	*GFLOPs* (t)	*mAP*@0.5:0.95 (%)	*FPS*
YOLO v8n	−	−	91.43	83.33	88.95	87	8.2	86.63	126
YOLO v8n-M	√	−	92.95	**92.23**	92.92	92	**5.8**	90.77	**239**
YOLO v8n-MPD	−	√	92.19	84.61	90.06	88	8.7	86.07	122
YOLO v8n-PP	√	√	**94.88**	89.11	**93.36**	**93**	6.1	**91.08**	215

Note: Bolded data are optimal values. And the “√” means the network improvement method.

Improvements to the algorithm include the optimization of the YOLO v8n model using a more efficient neural network structure, resulting in a significant reduction in computational effort while improving target detection accuracy and increasing the computational speed of the network model, with a maximum detection *FPS* of 239 fps. Compared to the unimproved YOLO v8n, the *P*, *R*, *mAP*@0.5, *F1*, *mAP*@0.5: 0.95, and *FPS* of YOLO v8n-M were raised by 1.52%, 8.9%, 3.97%, 5%, 4.14%, and 113 fps, and the *GFLOPs* was reduced by 2.4 t. It means that replacing Mobile-ViT for the network backbone can lead to a significant improvement in comprehensive network performance. After replacing the MPDIoU, the *P*, *R*, *mAP*@0.5, and *F1* of YOLO v8n-MPD were improved by 0.76%, 1.28%, 1.11%, and 1%, but the *GFLOPs* was also increased by 0.5 t, and its *mAP*@0.5:0.95, and the *FPS* decreased by 0.56% and 4 fps. The *P*, *R*, *mAP*@0.5, *F1*, *mAP*@0.5: 0.95, and *FPS* of YOLO v8n-PP were increased by 3.45%, 5.78%, 4.41%, 6%, 4.45%, and 89 fps, and the *GFLOPs* was decreased to 2.1 t.

By comparing the results of the ablation-style experiments, the seven evaluation metrics of the YOLO v8n-PP exhibit excellent performance in identifying the PV panel’s positional attitude. The four evaluation metrics *P*, *R*, *mAP*@0.5, *F1*, *mAP*@0.5: 0.95, and *FPS* reach their optimal values in this experiment, while the three evaluation metrics of *R*, *GFLOPs*, and *FPS* also demonstrate an elevated level of performance. The results of this ablation-style experiment fully validate the comprehensive performance advantages of the YOLO v8n-PP target detection model in PV panel position recognition.

### 4.2 Computer simulation results of path planning algorithms

**FIGURE 10:**
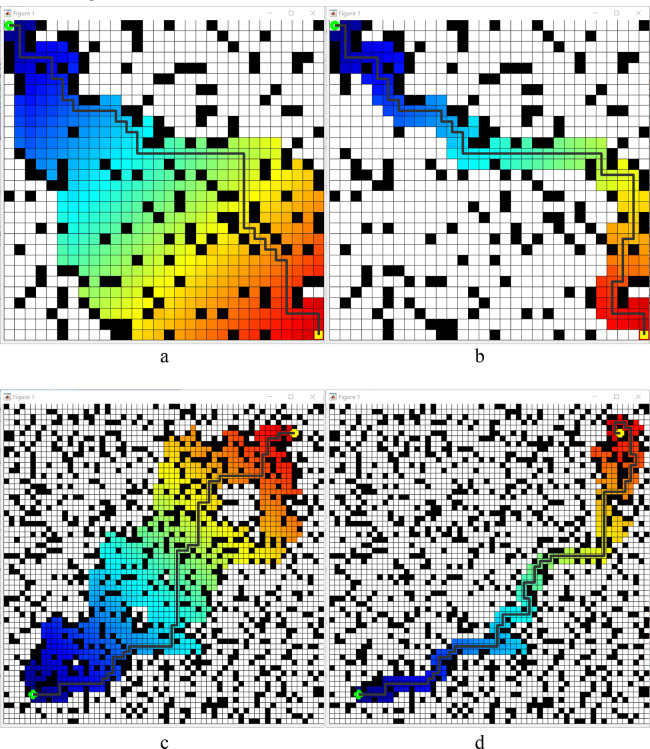
Comparison between before and after heuristic improvement. **(a)** 30 × 30 map results before improvement, **(b)** 30 × 30 map results after improvement. **(c)** 60 × 60 map results before improvement, **(d)** 60 × 60 map results after improvement. Those black pixels denote obstacles, while white denotes open space. The green plum dots in the lower left corner indicate the starting point, and the yellow dots in the upper right corner denote the goal point. Colored paths denote the area involved in path planning during movement. The distance from the starting point corresponds to the transition between warm and cold colours.

To compare the visualization results and running data more intuitively, this simulation experiment utilizes the MATLAB software. On the raster maps of two sizes, 30 × 30 and 60 × 60, the control variable method is used to unify the green circle marks on the left side as the starting point and the green circle mark on the right side as the endpoint. The starting point and end point positions are the same under the exact raster map specification. The number of obstacles generated accounts for 20% of the total number of squares, and the layout of obstacles remains unchanged. Simulation experiments of path planning are conducted for the heuristic functions before and after optimization, respectively, and the experimental results of the two cases are compared. The simulation results are shown in Figure 10.

Table 3 shows the results of the simulation experiments are as follows: under the 30 × 30 raster map, the optimized algorithm with heuristic function increases the length of the travelling path by 6.9%, but shortens the search time by 73.61%; under the 60 × 60 raster map, the optimized algorithm increases the length of the travelling path by 14.29%, but shortens the search time by 79.3%. These experimental results demonstrate that the A* algorithm optimised by the heuristic function aligns with the changing characteristics of path planning when the heuristic function h(n) is taken into account. Although it may not be the shortest traveling path, the path length increases slightly. The search time is significantly reduced, which verifies the validity of the heuristic function optimization in the A* algorithm.

**TABLE 3:** Compared the results of heuristic functions under different specifications of raster maps.

Map size (m^2^)	Method	Path length (m)	Search time (s)
30 × 30	original A*algorithm	58	4.51
	Heuristic optimization	62	1.19
60 × 60	original A*algorithm	98	8.60
	Heuristic optimization	112	1.78

**FIGURE 11:**
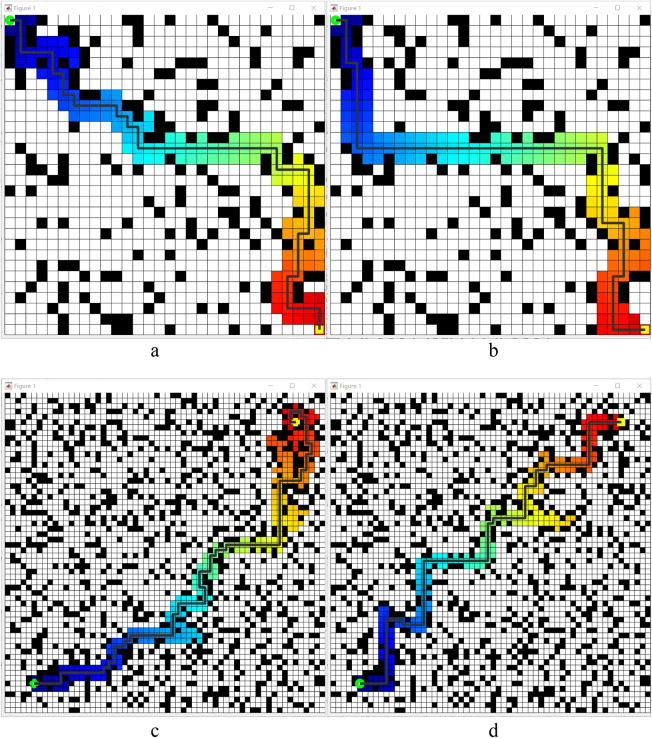
Corner smoothing optimization before and after. **(a)** 30 × 30 map results before improvement, **(b)** 30 × 30 map results after improvement. **(c)** 60 × 60 map results before improvement, **(d)** 60 × 60 map results after improvement. Those black pixels denote obstacles, while white denotes open space. The green plum dots in the lower left corner indicate the starting point, and the yellow dots in the upper right corner denote the goal point. Colored paths denote the area involved in path planning during movement. The distance from the starting point corresponds to the transition between warm and cold colours.

Table 4 shows the results of the simulation experiments are as follows: in a 30 × 30 raster map, the optimized algorithm with corner smoothing reduces the length of the travelling path by 3.23%, the search time by 44.54% and corners by 56.52%; in a 60 × 60 raster map, the optimized algorithm reduces the length of the travelling path by 10.71%, the search time by 19.1% and the Turn Number (*CN*, Corner Number, units places or p) by 55%. These experimental results show that the A* algorithm optimized by corner smoothing has reduced the path length. In contrast, the search time and the *CN* have been significantly reduced, which verifies the effectiveness of corner smoothing optimization for the A* algorithm shown in Figure 11.

**TABLE 4:** Comparison results of corner smoothing optimization under different specifications of raster maps.

Map size (m^2^)	Smoothing method	Path length (m)	Search time (s)	*CN* (p)
30 × 30	Without smoothing	62	1.19	23
	Corner smoothing	60	0.66	10
60 × 60	Without smoothing	112	1.78	40
	Corner smoothing	100	1.44	18

**FIGURE 12:**
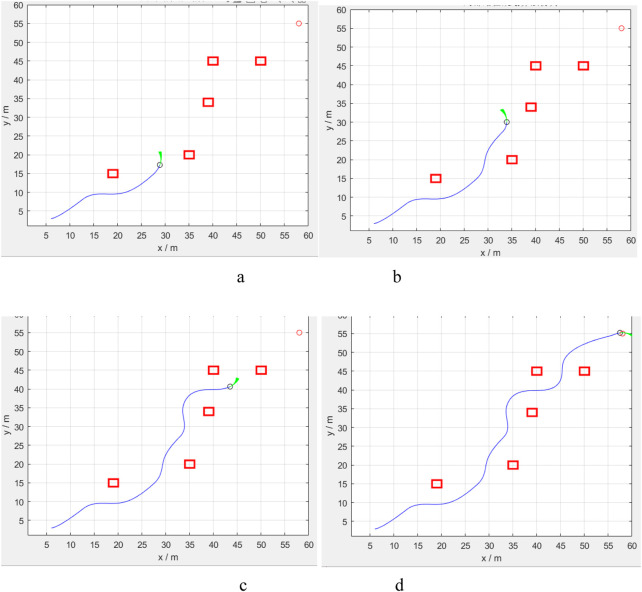
Simulation result of the local DWA path planning algorithm in MATLAB. **(a–d)** four key process of the optimised path planning method in this paper.

This simulation experiment is also based on the MATLAB, set the simulation range of 60 m × 60 m, the starting point and the end point are set as diagonal state to increase the path length, to more intuitively show the effect of the local path planning, the priority of the obstacle position is arranged in the vicinity of the diagonal straight line, and the simulation parameters are adjusted according to the speed setting of the robot: the maximum line speed is 5 m/s, the line acceleration is 0.2 m/s^2^, the maximum rotational speed is 20 rad/s, and the rotational acceleration is 50 rad/s^2^. The parameters of the evaluation function are set as follows: *α = 0.05*, *β = 0.1*, *γ = 0.*1. The simulation process is shown in Figure 12.

In the illustration, the red rectangular boxes represent obstacles, while the blue lines indicate the simulated robots’ traveling trajectories. The green ray beams illustrate the local path planning results simulated for various speed combinations. From Figure 12, the DWA (Dynamic Window Approach) algorithm effectively completes local path planning in the map, demonstrating the usefulness of DWA in the obstacle avoidance function.

### 4.3 Results of PV cleaning robots in a real environment

**FIGURE 13:**
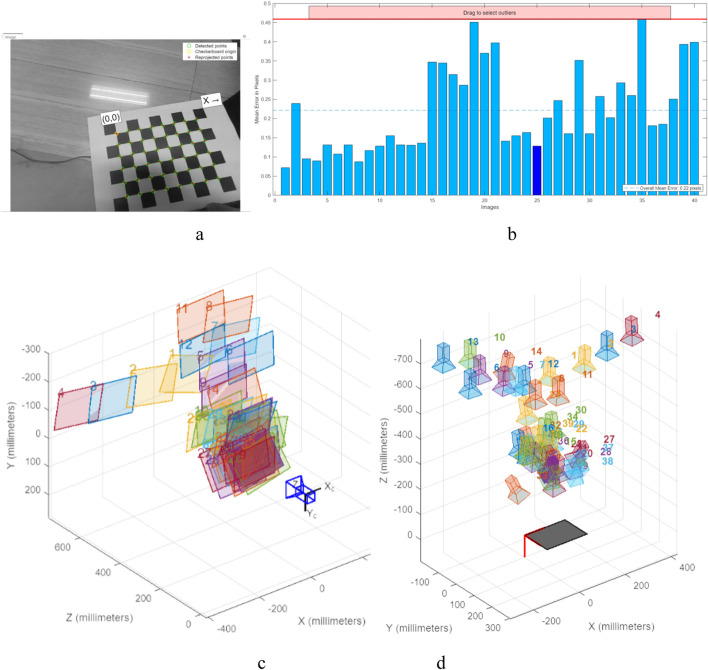
Calibration process and results in world coordinates. **(a)** the image data used for the calibration, **(b)** the error statistics during the calibration process. **(c)** is the camera-centered calibration of the viewpoints. **(d)** Calibration panel for the center calibration view.

Camera calibration is performed through MATLAB’s Camera Calibrator App to achieve the calibration of the robot’s coordinates to the world coordinates, to facilitate the effect of the simulation results in section 4.2, and the localization pathfinding results in section 3.3, to better understand the effects of the camera calibration and the cleaning of the robot. As shown in Figure 13, the image data of subfigure a was used for calibration, and after counting the error distribution of subfigure b, 40 groups of 0.5 pixel error data were all in the camera calibration error, and the corrective coordinate transformation was solved to obtain the robot’s internal reference matrix as $\left[\begin{array}{ccc} 477.6700 & 0 & 334.2009\\ 0 & 478.0829 & 238.3404\\ 0 & 0 & 1 \end{array}\right]$ The effective focal length is $f_{x}=477.67$ , $f_{y}=478.0829$ , The coordinates of the main focal point are $\left[\begin{array}{cc} 334.2009 & 238.3404 \end{array}\right]$ . The radial distortion coefficient is $\left[\begin{array}{cc} -0.0406 & 0.0727 \end{array}\right]$ . The tangential distortion coefficient is $\left[\begin{array}{cc} 0 & 0 \end{array}\right]$ , with an average reprojection error of 0.2214 pixels.

**FIGURE 14:**
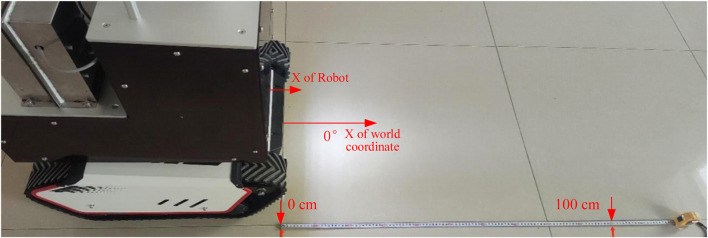
Calibration process and results for speed and direction.

Thereafter, the robot’s reference angle and reference speed must also be calibrated. The calibration principle is proportional calibration, which involves measuring the speed and reference coordinate direction, as shown in Figure 14. The initial position in the x-direction of the world coordinates is preset in the laboratory. The cleaning robot is allowed to start from the starting position by random forward movement and left rotation commands. It then returns to the original position and direction, stopping for the first time after 15 min. After this, the total distance *S* and the total amount of converted angle *θ* were counted. The measured movement deviation *s* and angular deviation *ω* are taken as the corrected data, which are then input into the angular velocity calibration program for error correction. Measure the moving deviation *s* and angular deviation *ω* for correction data and input them into the program for error correction, where $\frac{s}{S}=\frac{6}{100}$ is the velocity proportional deviation and $\frac{\omega }{\theta }=\frac{11}{360}$ is the angular proportional deviation.

**FIGURE 15:**
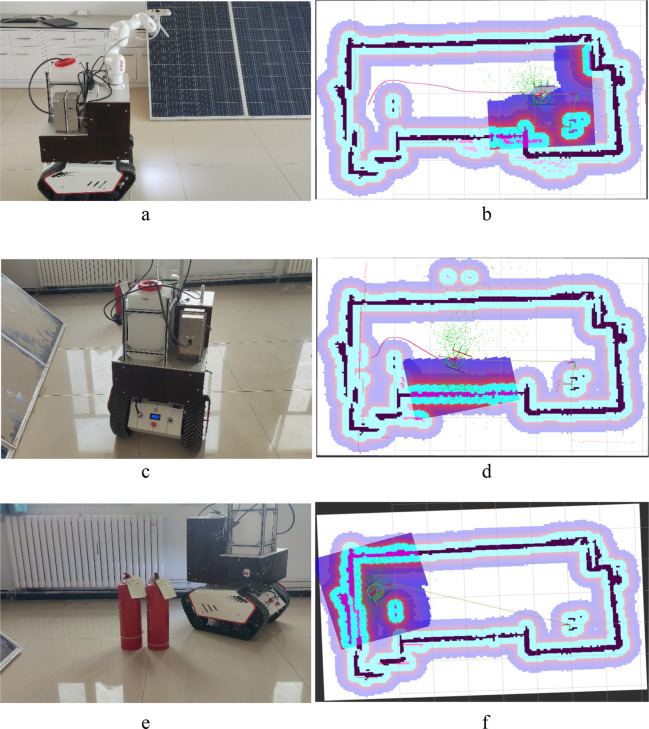
Test results of the robot in an indoor environment. **(a)** The map construction is for autonomous movement. **(b)** The map construction and path of **(a)**. **(c)** The path planning during autonomous movement. **(d)** The map construction and path of **(c)**. **(e)** The result of obstacle avoidance. **(f)** The map construction and path of the **(e)**.

Figure 15 illustrates the map construction and path planning process in the indoor laboratory environment, which evaluates the previously described map construction and path planning process. The a1 and a2 represent the map construction for autonomous movement, and b1 and b2 represent the path planning during autonomous movement. The c1 and c2 show the results of the obstacle avoidance process. As Figures 15a,b show, the path planning aims to obtain a straight line that avoids obstacles, rotates as little as possible, and moves efficiently, which is highly time-efficient. b to c shows that the process of obstacle avoidance can be conducted in both simulation (section 4.3) and laboratory environments. As the PV cleaning robot plans its path of travel and begins to move, dynamic obstacles quickly appear. When a dynamic obstacle appears in the local cost map, the PV panels cleaning robot quickly steers towards the PV panels to avoid colliding with the obstacle. It automatically enables local path planning, allowing the PV cleaning robot to replan its travel path to the target location and safely bypass obstacles. The calibration process and the calibration process above have a certain degree of accuracy.

**FIGURE 16:**
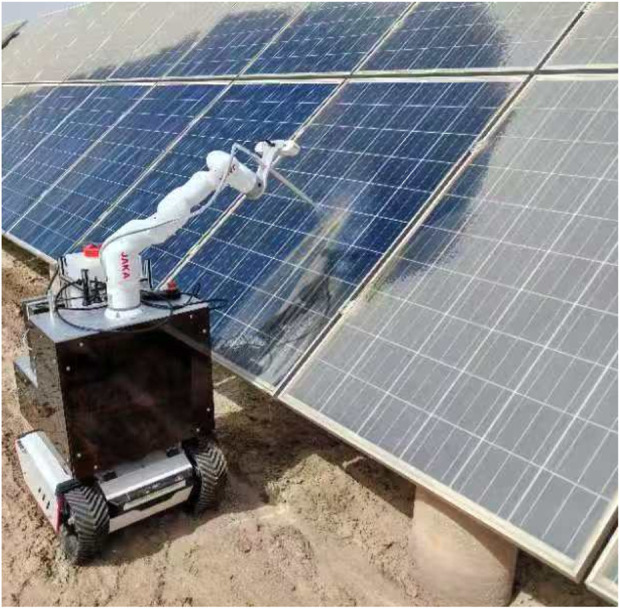
Results of PV panel cleaning in real-world environments.

The same setup and correction results were applied to the PV station cleaning test in the field environment. The test results are presented in Figure 16, demonstrating that the machine development in this paper has a specific cleaning effect and provides a reference value. Among them, the location DWA algorithm was modified to clean the front and back of the same row of 24 PV panels, resulting in an actual increase in cleaning speed of 23%. The results and the previous simulation test have a certain difference. Still, this difference is not contradictory to the point, because the algorithm improves the pathfinding process, specifically by completing the previous cleaning before moving on to the next cleaning target at the start of the process. The actual cleaning time also includes the operating time of the robotic arm, so the difference is also within reasonable limits.

Table 5 presents the results of a comparison between this study and several existing studies, including target detection methods, path planning algorithms, cleaning methods, and PV panel conditions. Among them, the Target detection method is used to indicate the detection method used in the implementation process, reflecting the functional accuracy and intelligence. The path planning algorithm reflects the automation in the execution of the work. The cleaning method refers to the practical approach used to clean, emphasising the efficiency of the work. PV panel condition is the universality of the method. The method described by Ma and Prabhakaran can achieve the detection of pollutants and is more universal in terms of the conditions of realisation; however, the subsequent cleaning process is not described. Jin and Yang can achieve cleaning without applying water, which is richer and better than the method in this paper, and Jin’s method is better than Yang and Riawan methods in terms of automation, although it uses the ACO algorithm to find the way. However, the Jin, Yang, and Riawan method does not identify the target of the work and is less accurate and intelligent than this paper’s method; however, the work is less automated than this paper’s method. Overall, in terms of functional accuracy, intelligence, and automation, this paper has certain advantages; however, subsequent research can be further strengthened in terms of cleaning and the universality of work conditions.

**TABLE 5:** Contrasts with several similar studies.

Method	Target detection method	Path planning algorithm	Cleaning method	PV panel condition
Ma method (Ma et al., 2022)	YOLO-PX	-	-	All
Prabhakaran method (Prabhakaran et al., 2022)	RMVDM	-	-	All
Grando robot (Grando et al., 2019)	-	-	Nozzles and water	With rail
Jin robot (Jin et al., 2024)	-	ACO algorithm	Both dry or with water	With rail
Yang robot (Yang et al., 2023)	-	-	Dry with brushes	Tilted and grounded
Riawan robot (Riawan et al., 2018)	-	-	Nozzles and water	Horizontal off-ground
Introduced robot	YOLO-v8n-PP	Location DWA	Nozzles and water	Tilted and grounded

## 5 Conclusion

Cleaning with an auto-robot is essential and a further trend for a PV station. This study enhanced the YOLO v8 network model to accurately detect objects, refined the DWA algorithm to plan paths, and developed a robot system for cleaning purposes. The main conclusions are as follows:

(1) A lightweight Mobile-ViT model incorporating a Self-Attention mechanism is utilized to enhance YOLO v8, yielding an accuracy of 91.08% and a processing speed of 215 fps.
(2) A* global path planning algorithm and DWA local path planning algorithm are improved, and the results in the simulation environment show that the time consumption decreases from 1.19 s to 0.66 s and the *CN* decreases from 23 to 10 p.
(3) The robot was debugged and calibrated in the PV station environment, and results showed that the robot could build a work map and clean without manual control, showing the rate increased by 23% after the improved algorithm.

The robot provided a reference for the engineering applications of deep learning, computer vision, and robot navigation. However, the robot in this paper requires further field tests and development to validate its effectiveness and advantages, such as multi-copter cooperation and collaboration with other entities (e.g., UAVs). For the detection of pollutants, further research can also identify their types, quantities, and locations, which would improve cleaning efficiency and energy utilisation rates.

## Data Availability

The raw data supporting the conclusions of this article will be made available by the authors, without undue reservation.

## References

[B1] Antonelli M. G. Beomonte Zobel P. De Marcellis A. Palange E. (2020). Autonomous robot for cleaning photovoltaic panels in desert zones. Mechatronics 68, 102372. 10.1016/j.mechatronics.2020.102372

[B2] Azouzoute A. Zitouni H. El Ydrissi M. Hajjaj C. Garoum M. Bennouna E. G. (2021). Developing a cleaning strategy for hybrid solar plants PV/CSP: case study for semi-arid climate. Energy 228, 120565. 10.1016/j.energy.2021.120565

[B3] Bergametti G. Forêt G. (2014). “Dust deposition. 179-200*179-200,” in Mineral dust: a key player in the Earth system. Editors Knippertz P. Stuut J. W. (Netherlands, Dordrecht: Springer).

[B37] Chen B. R. Hsu C. F. Lee T. T. (2023). Intelligent balancing and trajectory tracking control for unicycle robots. Int. J. Fuzzy Syst., 25, 2954-2968. 10.1007/s40815-023-01600-3

[B4] Chen Y. Zhou B. Ye D. Cui L. Feng L. Han X. (2023). An optimization method of deep transfer learning for vegetation segmentation under rainy and dry season differences in a dry thermal valley. Plants-Basel 12, 3383. 10.3390/plants12193383 37836123 PMC10574146

[B34] Cuenca A. Moncayo H. (2023). Geomagnetic navigation using Rao Blackwellized particle filter. In AIAA Scitech Forum and Exposition, 2023. American Institute of Aeronautics and Astronautics Inc. 10.2514/6.2023-1452

[B5] Gao R. Li A. (2012). Dust deposition in ventilation and air-conditioning duct Bend flows. Energ Convers. Manage 55, 49–59. 10.1016/j.enconman.2011.10.018

[B29] Gamani A. R. A. Arhin I. Asamoah A. K. (2024). Performance evaluation of YOLOv8 model configurations, for instance segmentation of strawberry fruit development stages in an open field environment. arXiv, arXiv:2408.05661.

[B6] Grando M. N. G. N. Maletz E. R. Martins D. Simas H. Simoni R. (2019). Robots for cleaning photovoltaic panels: state of the art and future prospects. Rev. Tecnol. Cienc. 137–150. 10.33414/rtyc.35.137-150.2019

[B35] Grisetti G. Stachniss C. Burgard W. (2005). Improving grid - based SLAM with Rao - Blackwellized particle filters by adaptive proposals and selective resampling. Proceedings of the IEEE International Conference on Robotics and Automation (ICRA). 10.1109/ROBOT.2005.1570566

[B33] Girshick R. (2015). Fast R-CNN. Proceedings of the IEEE International Conference on Computer Vision (ICCV). arXiv:1504, 08083.

[B7] Habib M. R. Tanvir M. S. Suhan A. Y. Vadher A. Alrashed A. Shawmee T. T. (2021). “Automatic solar panel cleaning system based on arduino for dust removal,” in 2021 International Conference on Artificial Intelligence and Smart Systems (ICAIS), Coimbatore, India, 25-27 March 2021, 1555–1558.

[B8] Hossain M. I. Ali A. Bermudez Benito V. Figgis B. Aïssa B. (2022). Anti-soiling coatings for enhancement of PV panel performance in desert environment: a critical review and market overview. Materials 15, 7139. 10.3390/ma15207139 36295209 PMC9609821

[B9] Hu S. Liu W. Wen H. Liu Z. Huang W. (2025). Numerical simulation of dust deposition on photovoltaic module surface based on multifactor fusion deposition mechanism. Sci. Total Environ. 959, 178327. 10.1016/j.scitotenv.2024.178327 39756298

[B10] Huang Y. P. Kshetrimayum S. Sandnes F. E. (2025). UAV-based automatic detection, localization, and cleaning of bird excrement on solar panels. IEEE Trans. Syst. Man, Cybern. Syst. 55, 1657–1670. 10.1109/tsmc.2024.3506533

[B11] Jin L. Meng G. Zheng H. Zhang J. Dong S. Deng X. (2024). Design and path planning method of control system for photovoltaic panel cleaning robot. J. Comput. 35, 175–190. 10.53106/199115992024083504013

[B12] Kshetrimayum S. Liou J. J. H. Huang Y. P. (2023). “A deep learning based detection of bird droppings and cleaning method for photovoltaic solar panels,” in 2023 IEEE International Conference on Systems, Man, and Cybernetics (SMC), Honolulu, United States, 01-04 October 2023, 3398–3403.

[B13] Li X. Li X. (2022). Development of following robot for supplying power to solar panel cleaning robot. Industrial Robot Int. J. robotics Res. Appl. 49, 88–95. 10.1108/ir-03-2021-0055

[B14] Licardo J. T. Domjan M. Orehovački T. (2024). Intelligent robotics—A systematic review of emerging technologies and trends. Electronics 13, 542. 10.3390/electronics13030542

[B36] Li X. Zhang D. (2023). Improved A* algorithm with DWA for dynamic obstacle avoidance in mobile robot navigation. J. of Intell. & Robot. Sys. 108(3), 1–15. 10.1007/s10846-023-01882-5

[B15] Liu X. Yue S. Li J. Lu L. (2021). Study of a dust deposition mechanism dominated by electrostatic force on a solar photovoltaic module. Sci. Total Environ. 754, 142241. 10.1016/j.scitotenv.2020.142241 33254918

[B31] Liu S. Qi L. Qin H. Shi J. Jia J. , “Path aggregation network for instance segmentation, ”2018 IEEE/CVF Conference on Computer Vision and Pattern Recognition, Salt Lake City, United States, 2018, 8759–8768. 10.1109/CVPR.2018.00913

[B30] Liu Y. Zhou X. , “Object detection algorithm for UAV aerial image based on improved YOLOv8,” 2024 5th International Conference on Electronic Communication and Artificial Intelligence (ICECAI), Shenzhen, China, 2024, 789–793. 10.1109/ICECAI62591.2024.10675035

[B16] Ma X. Han B. Xie H. Shan Y. (2022). An occlusion detection algorithm for small targets on the surface of photovoltaic modules based on deep learning. Proc. SPIE 12474, 8. 10.1117/12.2653504

[B17] Mariraj Mohan S. (2016). An overview of particulate dry deposition: measuring methods, deposition velocity and controlling factors. Int. J. Environ. Sci. 13, 387–402. 10.1007/s13762-015-0898-7

[B32] Mehta S. Rastegari M. (2021). Mobilevit: Light - weight, general - purpose, and mobile - friendly vision transformer. ArXiv preprint ArXiv: 2110.02178.

[B18] Milidonis K. Eliades A. Grigoriev V. Blanco M. J. (2023). Unmanned aerial vehicles (UAVs) in the planning, operation and maintenance of concentrating solar thermal systems: a review. Sol. Energy 254, 182–194. 10.1016/j.solener.2023.03.005

[B19] Mohd Nizam Ong N. A. F. Sadiq M. A. Md Said M. S. Jomaas G. Mohd Tohir M. Z. Kristensen J. S. (2022). Fault tree analysis of fires on rooftops with photovoltaic systems. J. Build. Eng. 46, 103752. 10.1016/j.jobe.2021.103752

[B20] Osmani K. Haddad A. Lemenand T. Castanier B. Ramadan M. (2020). A review on maintenance strategies for PV systems. Sci. Total Environ. 746, 141753. 10.1016/j.scitotenv.2020.141753 33027871

[B21] Patil P. A. Bagi J. S. Wagh M. M. (2017). “A review on cleaning mechanism of solar photovoltaic panel,” in 2017 International Conference on Energy, Communication, Data Analytics and Soft Computing (ICECDS), Chennai, India, 01-02 August 2017, 250–256.

[B22] Prabhakaran S. Annie Uthra R. Preetharoselyn J. (2022). Feature extraction and classification of photovoltaic panels based on convolutional neural network. Comput. Mater. Continua 74, 1437–1455. 10.32604/cmc.2023.032300

[B23] Qin N. Yu H. Wang P. Li X. Qiao X. Cheng W. (2024). Study on multiple influence mechanism of airflow-dust-gas coupling diffusion under large vortex flow technology. Build. Environ. 257, 111538. 10.1016/j.buildenv.2024.111538

[B24] Riawan I. P. G. Kumara I. N. S. Partha C. G. I. Nyoman Setiawan I. Santiari D. A. S. (2018). “Robot for cleaning solar PV module to support rooftop PV development,” in 2018 International Conference on Smart Green Technology in Electrical and Information Systems (ICSGTEIS), Bali, Indonesia, 25-27 October 2018, 132–137.

[B25] Sarode N. Ghugal P. Yadav S. Dantule S. Nandankar P. (2023). A comprehensive review on solar panel cleaning robot technologies. AIP Conf. Proc. 2753, 20018. 10.1063/5.0127800

[B26] Şevik S. Aktaş A. (2022). Performance enhancing and improvement studies in a 600 kW solar photovoltaic (PV) power plant; manual and natural cleaning, rainwater harvesting and the snow load removal on the PV arrays. Renew. Energ. 181, 490–503. 10.1016/j.renene.2021.09.064

[B27] Yang J. Zhao X. Gao Y. Guo R. Zhao J. (2023). Research on mechanism design and kinematic characteristics of self-propelled photovoltaic cleaning robot. Appl. Sci. 13, 6967. 10.3390/app13126967

[B28] Yuan Y. Zhou M. (2024). “Research on the intelligent control system of aerial robots,” in Advances and challenges in advanced unmanned aerial systems: selected contributions of ICAUAS 2023. Editors Liu Z. Li R. He X. Zhu Z. (Singapore: Springer Nature Singapore), 65–75.

